# Detector-device-independent quantum secret sharing with source flaws

**DOI:** 10.1038/s41598-018-23876-4

**Published:** 2018-04-10

**Authors:** Xiuqing Yang, Kejin Wei, Haiqiang Ma, Hongwei Liu, Zhenqiang Yin, Zhu Cao, Lingan Wu

**Affiliations:** 1 College of Science, Inner Mongolia University of Technology, Hohhot, 010051 China; 2 Guangxi Key Laboratory for Relativistic Astrophysics, School of Physics Science and Technology, Guangxi University, Nanning, 530004 China; 3 School of Science, Beijing University of Posts and Telecommunications, Beijing, 100876 China; 4 Key Laboratory of Quantum Information, University of Science and Technology of China, Hefei, 230026 China; 5 Center for Quantum Information, Institute for Interdisciplinary Information Sciences, Tsinghua University, Beijing, 100084 China; 6 Laboratory of Optical Physics, Institute of Physics, Chinese Academy of Sciences, Beijing, 100080 China

## Abstract

Measurement-device-independent entanglement witness (MDI-EW) plays an important role for detecting entanglement with untrusted measurement device. We present a double blinding-attack on a quantum secret sharing (QSS) protocol based on GHZ state. Using the MDI-EW method, we propose a QSS protocol against all detector side-channels. We allow source flaws in practical QSS system, so that Charlie can securely distribute a key between the two agents Alice and Bob over long distances. Our protocol provides condition on the extracted key rate for the secret against both external eavesdropper and arbitrary dishonest participants. A tight bound for collective attacks can provide good bounds on the practical QSS with source flaws. Then we show through numerical simulations that using single-photon source a secure QSS over 136 km can be achieved.

## Introduction

Quantum secret sharing (QSS) is a multiparty protocol^1–4^ to distribute a secret to a network of players, each of whom is allowed to access a share of the secret. It is possible for them to obtain the final key only if they all say yes. Secret sharing has many useful applications in network-based scenario, ranging from online auctioning, remote voting, master key of nuclear missile to multiparty secure computation. One of the desirable protocols for QSS is that three parties Alice, Bob and Charlie share the GHZ state $|{{\rm{\Phi }}}_{0}^{\pm }\rangle =\mathrm{(|000}\rangle \pm \mathrm{|111}\rangle )/\sqrt{2}$. Each of them randomly perform a projection measurement on their own photons either along *X* basis or along *Y* basis. The results of the three members in some measurement basis have perfect correlation and therefore can be used for QSS. As Charlie will obtain a deterministic outcome, e.g., ${X}_{c}={X}_{a}\oplus {X}_{b}$, she can force Alice and Bob to share the secret key with her only after performing a cooperative operation.

Compared with quantum key distribution (QKD), the security analysis of the multiparty protocol is complicated and its security has been challenged over time. The deviations between the components used for experimental realizations and the models in the security proof have led to information leaking to the eavesdropper. For example, Although it was claimed that a QSS procedure can be securely implemented using GHZ state^3^, we find out it is potentially vulnerable to a double blinding-attack by exploiting controllability of single-photon avalanche-photodiode-based detectors of both Alice and Bob instead of one^5^. That is, Eve intercepts the photon sent by Charlie and then performs measurements in random basis, as Alice (Bob) would have done it. In order to hide her presence, Eve blinds Alice’s (Bob’s) detectors so that the detector click only when the signal with peak power above a threshold *P*_*th*_ is reaching. After each detection, Eve forwards to Alice (Bob) a bright pulse corresponding to her measurement result, which deterministically gives Eve the same result as Alice’s (Bob’s) if their bases are identical, and no result at all if not. After Eve discards the few faked state in the reconciliation between Alice and Bob, she has the same bit value as theirs.

For practical QKD, the most general threats seem to be introduced by exploiting controllability of measurement devices including basis-choice apparatuses and single photon detector (SPD). Security threats like this are more implementation-friendly, of which time-shift attack^6^, after-gate attack^7^, blinding attack^5^ and laser damage^8^ attack have been demonstrated successfully. Scientists have put much effort towards building loophole-free QKD systems with untrusted devices. One important approach is to develop device independent protocols. Among them, the measurement-device-independent quantum key distribution (MDI-QKD)^9^ is automatically immune to all side-channel attacks by allowing Eve to fully control the measurement device. Recently, a detector-device-independent quantum key distribution (DDI-QKD)^10^ has been to proposed to exhibit a connection between the MDI-QKD and conventional BB84-like protocol. Although DDI-QKD is not precisely as secure as MDI-QKD, it may possess a high key rate of conventional QKD and exceed the performance and practicality of MDI-QKD in circumventing detector side channels. One crucial assumption behind DDI-QKD is that the linear optical elements of Bell-state measurement (BSM) must be trusted or some trustworthiness to the untrusted BSM device is required^11^.

Compared with QKD, both theoretical and experimental works on real applications in secure multiparty communication, such as QSS^12,13^, are rare. Following a similar spirit to DDI-QKD, we propose a detector-device-independent quantum secret sharing (DDI-QSS) protocol against all detector side-channels. The DDI-QSS protocol is designed to distribute a secret only when a separable state will never be wrongly identified as an entangled one based on measurement-device-independent entanglement witness (MDI-EW)^14,15^. We remark that source flaws are a serious concern in practical communication, not only in decoy-state QKD implementation but also in multiparty tasks including the fascinating MDI-QSS. For this reason, until now, the practicality of long-distance multiparty communication tasks has remains unknown. What we propose here is an entirely new approach to distributing a secret to the two authorized parties over long distances despite the source flaws. We obtain a condition on secure key against general attacks of an eavesdropper and cheating methods of dishonest players, and we prove that its security is independent of source error.

## Measurement-device-independent entanglement witness

It is known that there always exist an MDI-EW for any entangled state with untrusted measurement, even if the measurement devices are controlled by Eve. There are two situations in the so-called semiquantum nonlocal games. One would be a case where Alice and Bob want to verify their entanglement themselves. They prepare some ancillary state pairs (*τ*_*s*_, *ω*_*t*_), and send them along with the bipartite state *ρ*_*AB*_ to Eve. Eve performs two Bell-state measurement(BSMs) on *ρ*_*A*_(*ρ*_*B*_) and *τ*_*s*_(*ω*_*t*_), and gets some classical output *a* and *b*. For a bipartite entangled state *ρ*_*AB*_, we always find a conventional entanglement witness *W* decomposed in the form

1 $$W=\sum _{s,t}\,{\beta }_{s,t}{\tau }_{s}^{T}\otimes {\omega }_{t}^{T},$$

with real coefficients *β*_*s*,*t*_ such that *tr*(*Wρ*_*AB*_) < 0, while *tr*(*Wσ*_*AB*_) ≥ 0 for all separable states *σ*_*AB*_. In the MDI-EW design, an witness detecting the entanglement of *ρ*_*AB*_ can be obtained by

2 $$I({\rho }_{AB}^{v})=\sum _{s,t}\,{\beta }_{s,t}^{+,+}p(+,+|{\tau }_{s},{\omega }_{t}),$$

where ${\beta }_{s,t}^{+,+}$ = *β*_*s*,*t*_ and the probability distribution *p*(+, +|*τ*_*s*_, *ω*_*t*_) is obtained by projecting onto the maximally entangle state $|{{\rm{\Phi }}}^{+}\rangle =\mathrm{(|00}\rangle +\mathrm{|11}\rangle )/\sqrt{2}$. Mathematically, $I({\rho }_{AB}^{v})$ is always positive for all separable states, but is negative for certain entangled states. We show that Alice and Bob can obtain secure key in a MDI-EW scenario. We prove the security of practical QKD system is independent of source flaws.

Another situation would be a case where the third party wants to be convinced two untrusted members share entanglement. For example, Charlie who is in the parent company wants to identify whether an bipartite state *ρ*_*AB*_ is entangled in an untrused scenario. Similar to the above case, Charlie sends quantum state (*τ*_*s*_, *ω*_*t*_) to Alice and Bob, who perform BSMs on *ρ*_*A*_(*ρ*_*B*_) and *τ*_*s*_(*ω*_*t*_). Note that in both cases, it requires that the input states must be perfect. When using imperfect states, the MDI-EW could wrongly conclude a separable state to be entangled due to imperfect input state and thus indeed leads to an erroneous estimation of $I({\rho }_{AB}^{v})$.

## Protocol

The task of secret of sharing is as follows. Charlie, the president of a bank, wants to give access to a vault to two vice presidents, Alice and Bob. Instead of giving the combination to anyone individual, Charlie transmit a qubit string to Alice and Bob. It may be desirable to distribute information in such a way that using the MDI-EW Charlie detects an entangled state *ρ*_*AB*_ and perfect correlations among Alice, Bob and Charlie are obtained for QSS. There exists an equivalence between the security of the QSS and the success of the EW because it is crucial for Charlie to prove that a given state is entangled or not. Originating from this analogy, we propose a practical QSS protocol with untrusted detectors used in an EW process. However, a crucial assumption for the present protocol is that the linear optical elements of BSM inside the receivers’ laboratories must be trusted. That is measurement device is assumed to be a well-defined projective measurement acting on the two photons. This is indeed similar to the case in the concept of DDI-QKD, which requres perfect linear optical elements of BSM.

**Figure 1:**
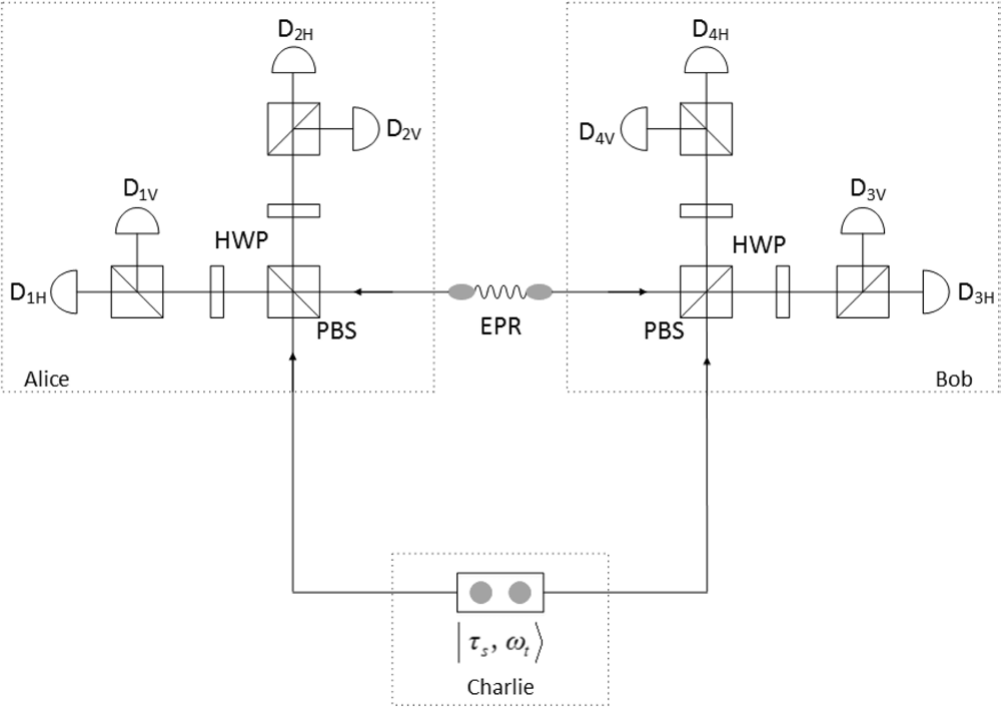
The schematics of the experimental setup for the DDI-QSS. Charlie prepare single-photon state pairs |*τ*_*s*_, *ω*_*t*_〉 as the signal states. The Werner state preparation setup consist of photon pairs generation by spontaneous parametric down conversion (SPDC). The experimental setup for Bell analysers consist of polarizing beam splitter (PBS) and half-wave plate (HWP) at 22.5°. All the photons are detected by sing-photon detector D.

In the following, we design DDI-QSS scheme in a MDI-EW process. As shown in Fig. 1, Charlie prepares single-photon input state pairs $$({\tau }_{s},{\omega }_{t})\in \{|H,H\rangle ,|H,V\rangle ,|V,H\rangle ,|V,V\rangle ,|D,D\rangle $$, $$|D,\tilde{D}\rangle ,|\tilde{D},D\rangle ,|\tilde{D},\tilde{D}\rangle ,|L,L\rangle ,$$$$|L,R\rangle ,|R,L\rangle ,|R,R\rangle \}$$, from spontaneous parametric down-conversion (SPDC) processes. Charlie sends quantum states pairs, *τ*_*s*_ to Alice and *ω*_*t*_ to Bob, who in this scenario do share some certain quantum states. More precisely, we consider the two-qubit Werner state

3 $${\rho }_{AB}^{v}=v|{{\rm{\Psi }}}^{-}\rangle \langle {{\rm{\Psi }}}^{-}|+\mathrm{(1}-v)I\mathrm{/4,}$$

with the visibility *v* ∈ [0, 1] and the singlet state $|{{\rm{\Psi }}}^{-}\rangle =\mathrm{(|01}\rangle -\mathrm{|10}\rangle )/\sqrt{2}$. Alice and Bob project their part of shared state together with these input states onto the maximally entangle state $|{{\rm{\Phi }}}^{+}\rangle =\mathrm{(|00}\rangle +\mathrm{|11}\rangle )/\sqrt{2}$ or $|{{\rm{\Phi }}}^{-}\rangle =\mathrm{(|00}\rangle -\mathrm{|11}\rangle )/\sqrt{2}$. To implicitly express MDI-EW in the form of Eq. (2), we define a possible decomposition for an EW

4 $$W=\frac{1}{2}\mathbb{1}-|{{\rm{\Psi }}}^{-}\rangle \langle {{\rm{\Psi }}}^{-}|,$$

on the basis of three Pauli matrices. Then we get

5 $$\begin{array}{rcl}I & = & \frac{2}{6}P(+,+|H,H)-\frac{1}{6}P(+,+|H,V)\\  &  & -\frac{1}{6}P(+,+|V,H)+\frac{2}{6}P(+,+|V,V)\\  &  & +\frac{2}{6}P(+,+|D,D)-\frac{1}{6}P(+,+|D,\tilde{D})\\  &  & -\frac{1}{6}P(+,+|\tilde{D},D)+\frac{2}{6}P(+,+|\tilde{D},\tilde{D})\\  &  & +\frac{2}{6}P(+,+|L,L)-\frac{1}{6}P(+,+|L,R)\\  &  & -\frac{1}{6}P(+,+|R,L)+\frac{2}{6}P(+,+|R,R).\end{array}$$

With the MDI-EW method, Charlie will allow two legitimate users, Alice and Bob, to jointly share the secret key with her. Entanglement witness is estimated with three different bases, but the secret key is extracted in the *X* basis. Charlie encodes |Φ^+^〉 as 1 and |Φ^−^〉 as 0, while Alice and Bob encodes |*D*, *D*〉 ($|\tilde{D},\tilde{D}\rangle$) as 0 and $|D,\tilde{D}\rangle$ ($|\tilde{D},D\rangle$) as 1. In each quantum transmission, Charlie prepares state pairs in a basis which makes it easy to detect entanglement and distribute a secret with high efficiency. Compared to similar protocols, it does not require announcing basis choice and discarding those data in different basis. When quantum state they share is entangled, we can obtain the perfect correlation among Alice, Bob and Charlie in some successful outcomes. As illustrated in Table 1, the key is extracted from the data of *X* basis except for those data used to identify entanglement. It is clear that after Charlie split a message into two parts, neither Alice nor Bob can it but they together can.

**Table 1:** Correlations among Alice, Bob and Charlie in the *X* Basis.

Alice	Bob	Charlie
|Φ^+^〉	|Φ^+^〉	$$|D,\tilde{D}\rangle $$ or $$|\tilde{D},D\rangle $$
|Φ^+^〉	|Φ^−^〉	|*D*, *D*〉 or $$|\tilde{D},\tilde{D}\rangle $$
|Φ^−^〉	|Φ^+^〉	|*D*, *D*〉 or $$|\tilde{D},\tilde{D}\rangle $$
|Φ^−^〉	|Φ^−^〉	$$|D,\tilde{D}\rangle $$ or $$|\tilde{D},D\rangle $$

In our scenario, based on the MDI-EW perfect correlations among Alice, Bob and Charlie are obtained, and therefore can be used for QSS without trusting their detectors. Considering some attacks on QKD based on the detection efficiency loophole, the detectors used by Bob will report no detection, or have a low detection efficiency when Eve’s and Bob’s setting differ. Similarly, Eve wants to determine a Bell state projection |Φ^+^〉 by remotely influencing the influencing the detectors, so that Bob is only to allowed to produce a specified output, maybe double-click *D*_3*H*_ and *D*_4*H*_. As a result, in this run the other possible output *D*_3*V*_ and *D*_4*V*_ for |Φ^+^〉 can not be observed. This attack is simialr to time-shift attack on QKD, however, it could not break the QSS system. We emphasize that the MDI-EW is not prone to any detection loophole, contrary to standard EW, and the present protocol is naturately immune to attacks by exploiting detection efficiency loophole, including the overwhelming blinding attack. Importantly, Alice, Bob and Charlie can obtain an information-theoretically secure key in an entanglement witness process.

## Security analysis

### Collective attacks

For charlie the purpose of QSS protocol can be recognized as an equivalent one to verify entanglement, which is also the purpose of entanglement witness. We note that there are two parameters, the value of entanglement witness *I* and the error rate in the *X* basis *e*_*x*_ that used to quantify Eve’s information. Without loss of generality, we can suppose the bipartite state for Alice and Bob is two-qubit Bell-diagonal state^16–18^ ${\rho }_{AB}={\lambda }_{1}|{{\rm{\Phi }}}^{+}\rangle \langle {{\rm{\Phi }}}^{+}|+{\lambda }_{2}|{{\rm{\Phi }}}^{-}\rangle \langle {{\rm{\Phi }}}^{-}|+{\lambda }_{3}|{{\rm{\Psi }}}^{+}\rangle \langle {{\rm{\Psi }}}^{+}|+{\lambda }_{4}|{{\rm{\Psi }}}^{-}\rangle \langle {{\rm{\Psi }}}^{-}|$, with ${\sum }_{i}\,{\lambda }_{i}=1$. The reason is as follows. Due to symmetry, we should have obtained the correlations *P*(1, 1) = *P*(0, 0) and *P*(0, 1) = *P*(1, 0), where *P* is the probability to get a pair of *a*, *b* ∈ {0, 1} with respect to three basis. Were this not the above symmetric scenario, we can apply a similar idea to the DDI-QSS and agree on permuting and flipping randomly a chosen half of their bit pairs^19,20^. The bit flip procedure would not change the above parameters, and would be public in classical communication. The symmetry of this protocol implies they can bound Eve’s information by restricting to collective attacks such that the initial quantum state ${\rho }_{AB}^{v}$ can be transformed into a Bell-diagonal state^21^. Following the QKD protocol^16^, for collective attacks Eve’s information is given by the Holevo quantity $\chi (A|E)=\chi (B|E)\le h(4I+\tfrac{1}{2})$. With the observed parameters *I* and *e*_*x*_, the key rate is

6 $$r\ge 1-h({e}_{x})-h(4I+\tfrac{1}{2}),$$

where *h* is the binary entropy.

It is worth noting that dut to the imperfect states, the MDI-EW may consider some biseparable states as an entangled one. In the same manner, we repeat an argument for the DDI-QSS: the security in practical system is source-error-independent. To quantify the quantum states, the states to Alice can be written as |*α*_1_〉, |90° + *α*_2_〉, |45° + *α*_3_〉, |−45° + *α*_4_〉, $|{e}^{i\mathrm{(45}^\circ +{\alpha }_{5})}\rangle$ and $|{e}^{i(-45^\circ +{\alpha }_{6})}\rangle$, with modulation error *α*_1_, *α*_2_, *α*_3_, *α*_4_, *α*_5_ and *α*_6_. Meanwhile, the situation is similar for Bob’s states. For a Bell-diagonal state, we thus obtain *I*′ > *I*. This implies the perfect sources witness entangled states in the worst case compared to the flawed sources. The secret key rate in practical QSS system thus can be give by Eq. 6. Compared with the postselected GHZ states scheme^12^, we obtain a long distribution distance among Alice, Bob and Charlie for practical QSS with the source flaws.

### Participant attacks

We point out here using the MDI-EW method, we provide condition on the secure key against both external eavesdropper and dishonest participants. The main idea in our approach, to deal with arbitrary cheating strategies is that Charlie wants to identify whether the two untrusted parties, Alice and Bob, share entanglement according to input and output of the BSM. It is a natural assumption that the dealer Charlie is considered to be trusted party with trusted device.

Suppose that Bob is a dishonest player, he expects to access Alice’s secret by himself entirely bypassing the aforementioned collaboration with Alice. A most general cheating strategy for Bob would be the below attack. First, he performs the BSM using his local measurement *ω*_*t*_, *b*. Meanwhile, he also intercepts the signal sent from Charlie to Alice and performs Bell state measurement *τ*_*s*_, *a*. Second, according to detection outcome *a* he tells Alice’s device to produce specified value as outputs so that the procedure for secret sharing deviates from the protocol. Bob can therefore determine Alice’s value based on the following rules: If Bob obtain |Φ^+^〉 or |Φ^−^〉, he will send the corresponding single-photon state pairs |*D*, *D*〉 or $|D,\tilde{D}\rangle$ to Alice. In other cases, he will send state pairs |*H*, *H*〉, |*V*, *V*〉 or no detection to Alice. Receiving the state pairs, Alice’s detection probability is only 50% for |Φ^+^〉 and |Φ^−^〉. For this reason, Bob’s BSM probability is twice as big as Alice’s. However, Bob can carefully control the announcement rate to make it compatible with Alice’s results so that Bob can conceal his cheating in a postselection process.

The cheating strategy discussed above can be partly prevented by a modified protocol so that Bob hardly simulate a entanglement witness based on three bases. The DDI-QSS protocol uses the data in the *X* basis to extract secure key and the *Z*, *Y* basis to test entanglement. Hence, Alice can choose a small fraction of *Z*, *Y* basis so that it is sufficient to evaluate the entanglement witness. After Alice and Bob announce the measurement results, Charlie calculates the BSM probability corresponding to three basis. When the statistical result deviates a desired range, they will abort it. As a result, Bob’s cheating strategy is inefficient to generate a key by himself.

### Simulation

We give a numerical simulation using an ideal single-photon source prepared by Charlie and one EPR state (singlet) prepared by an eavesdropper. We consider the conditions of detection from the paper^22^ with a detection efficiency of *η* = 0.1 and a dark count rate *d* = 10^−5^, whereas here we consider a fiber-based channel. Then the probability for a detector to record a photon through transmission distance *l* is *p*_*ρ*_ = *η* 10^−*αl*/10^, with a loss coefficient *α* = 0.2 dB/km. The polarization misalignments and losses of the transmissions of the four quantum channels (i.e., Charlie to Alice and Bob, EPR source to Alice and Bob) are assumed to be identical.

**Figure 2:**
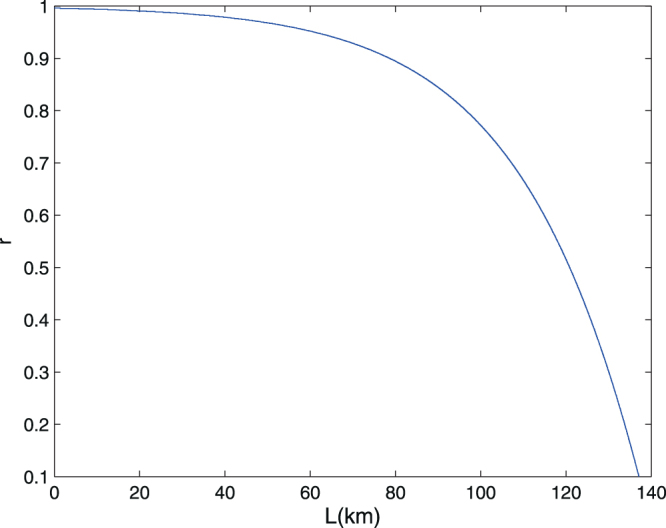
Lower bound on the key rate (per sifted key bit) versus fiber channel transmission from Charlie (EPR source) to Alice (Bob). A secret key rate with perfect single-photon states is illustrated. We show the simulation result of four identical quantum channels for the given parameters.

For post-processing, Charlie evaluates the data of *I* and the data of *e*_*x*_ separately. We consider actual detection condition, in which the probability corresponding to two successful Bell-state measurement in three bases is ${p}_{d}={p}_{\rho }^{3}(1-{p}_{\rho })d{(1-d)}^{4}$ + $6{p}_{\rho }^{2}{(1-{p}_{\rho })}^{2}{d}^{2}{(1-d)}^{4}$ + $8{p}_{\rho }{(1-{p}_{\rho })}^{3}{d}^{3}{(1-d)}^{4}$ + $16{(1-{p}_{\rho })}^{4}{d}^{4}{(1-d)}^{4}$. Here, the first item represents three-photons click and a dark count, the second describes two-photons and two dark counts, the third denotes one photon and three dark counts, and the fourth is four dark counts. Otherwise, the probability to obtain two BSM results accounting for four photons in the *X* basis is $p=\frac{1}{4}{p}_{\rho }^{4}{(1-d)}^{4}$. In the *X* basis, an error corresponds to a projection into |Φ^−^Φ^−^〉 or |Φ^+^Φ^+^〉 when Charlie prepare the same states, or, into |Φ^+^Φ^−^〉 or |Φ^−^Φ^+^〉 with orthogonal states. The QBER in the *X* basis can be written as ${e}_{x}=\frac{1}{2}\frac{{p}_{d}}{{p}_{d}+p}$. Likewise, for Alice and Bob the probability to obtain a successful projection into |Φ^+^Φ^+^〉 in three bases is $p(H,H)=$ $p(V,V)=p(D,D)$ =$p(\tilde{D},\tilde{D})=p(L,L)=p(R,R)=\frac{1}{4}{p}_{d}$,and$p(H,V)=p(V,H)=p(D,\tilde{D})=$ $p(\tilde{D},D)$ $p(L,R)=p(R,L)$= $\frac{1}{8}{p}_{\rho }^{4}{(1-d)}^{4}+p(H,H)$. Given these, one can calculate the value of *I* in Eq. (6). The resulting numerical simulation of the secret key rate are shown in Fig. 2. We would like to mention that the realization of our idea requires expanding the distance between entangled particles. On the basis of present fiber and detector technology, it has shown that the distance for distributing entanglement is limited to the order of 100 km^23^. Our result demonstrates the feasibility of quantum communication using entangled pairs with standard optical components.

What we take into consideration here is the signal state Charlie prepares must be single-photon state. A secure key can finnally be distilled with some practical sources only if we know the lower bound of the fraction of those raw bits contributed solely by the single-photon state components. We know that with the help of decoy-state method, one can perform QSS with weak coherent state sources using phase postselection technique or quantum nondemolition measurement technique^12^. Here in a model with one PDC source in the middle, it would be interesting to explore whether the decoy-state method will accurately and efficiently verify such a bound.

## Discussion

To conclude, We propose a double blinding-attack on a QSS protocol based on GHZ state. we have shown that using the MDI-EW method one can securely distribute a key between the two agents against all detector side channels. We extend the trusted device boundary in both sides to include the linear optical elements of BSM, except that the single-photon detectors are untrusted. We show that it is unconditionally secure against both an eavesdropper and dishonest players. For collective attacks, we obtain a bound on the key rate with source flaws. With the chosen parameters, we realize a DDI-QSS using single-photon sources over a distance of about 136 km from Charlie to Alice (Bob). It is expected that by following our proposal, a long-distance quantum secret sharing can be achieved experimentally.
